# P-36. Evaluating outcomes for short (≤ 10 days) versus long (> 10 days) duration of therapy for Gram-negative bacteremia in kidney transplant recipients

**DOI:** 10.1093/ofid/ofaf695.265

**Published:** 2026-01-11

**Authors:** Kirby An, Sumeet Jain, Patricia Saunders-Hao, Nicholas Jandovitz, Esther Benamu, Tungming Leung

**Affiliations:** North Shore University Hospital, Oakland Gardens, NY; North Shore University Hospital, Oakland Gardens, NY; North Shore University Hospital, Oakland Gardens, NY; North Shore University Hospital, Oakland Gardens, NY; Transplant Institute, Northwell Health, Manhasset, New York; Northwell Health, Manhasset, New York

## Abstract

**Background:**

Gram-negative bacteremia in solid organ transplants is a common complication and has been associated with morbidity and mortality. Despite the shift to shorter durations of therapy for Gram-negative bacteremia in recent literature, the question of duration for Gram-negative bacteremia in kidney transplant recipients (KTR) remains.

**Figure 1:**
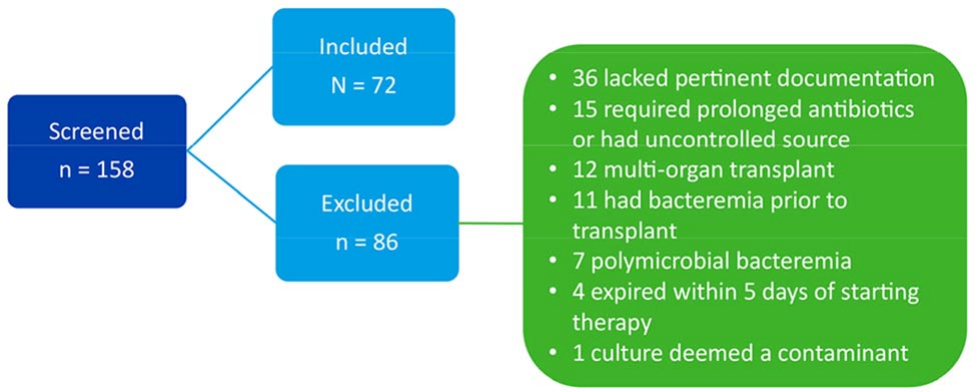
Enrollment

**Table 1:**
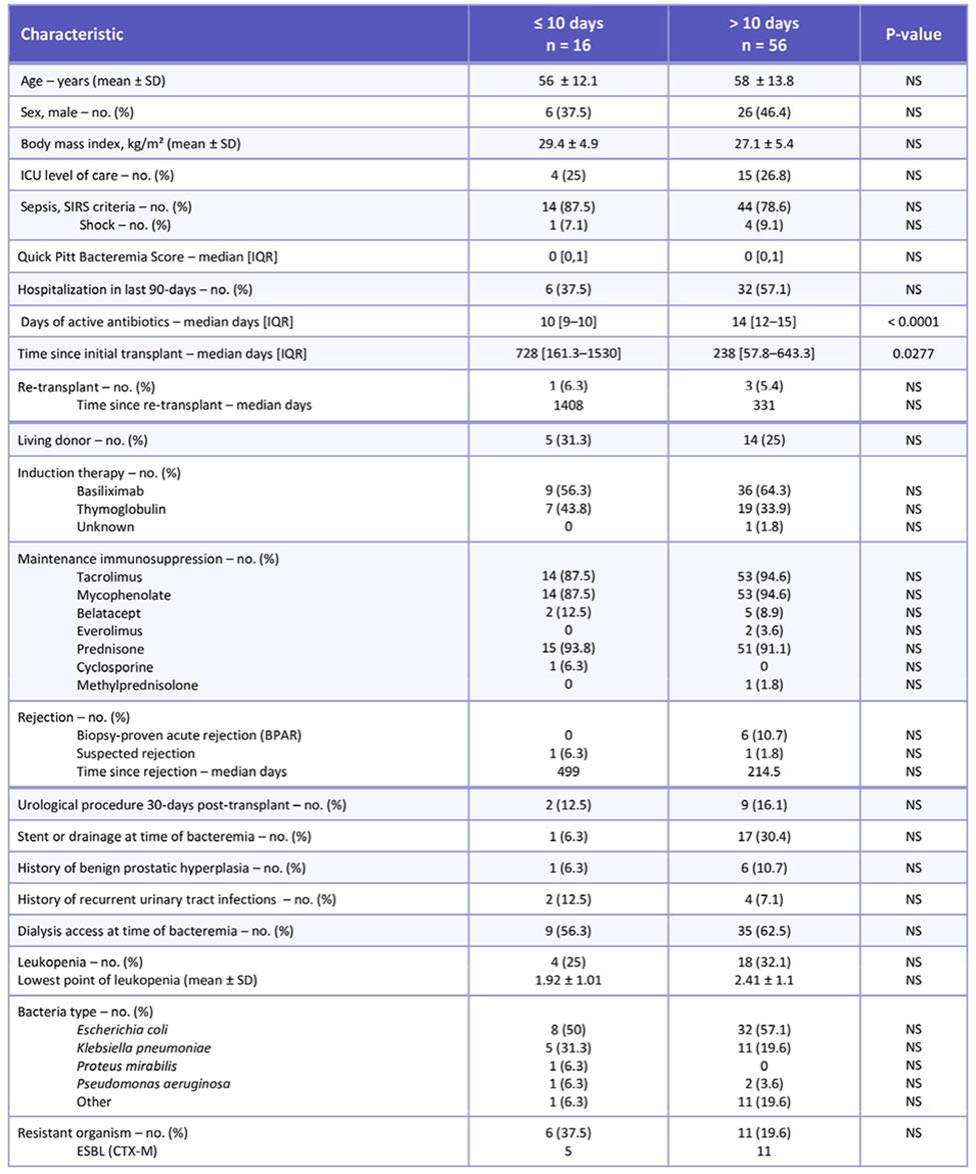
Baseline characteristics

**Methods:**

This IRB exempt, retrospective observational chart review evaluated adult KTRs with Gram-negative bacteremia who received therapy for ≤ 10 days vs. > 10 days between January 2016 to June 2024. Patients were included if they received active antibiotics for at least 5 days. Patients were excluded if they had a polymicrobial bacteremia, bacteremia secondary to infections requiring prolonged antibiotics, concomitant infection requiring antibiotics at the time of diagnosis of the Gram-negative bacteremia, uncontrolled source of infection, or history of multiorgan transplant. The primary outcome is a composite of 90-day mortality or microbiologic relapse. Secondary outcomes include a composite of 30-day mortality or relapse, hospital length of stay (LOS), development of multi-drug resistant (MDR) bacteria, and transplant rejection. We performed a subgroup analysis of our 90-day composite outcome in those transitioned to oral (PO) vs. continued intravenous (IV) antibiotics.

**Figure 2:**
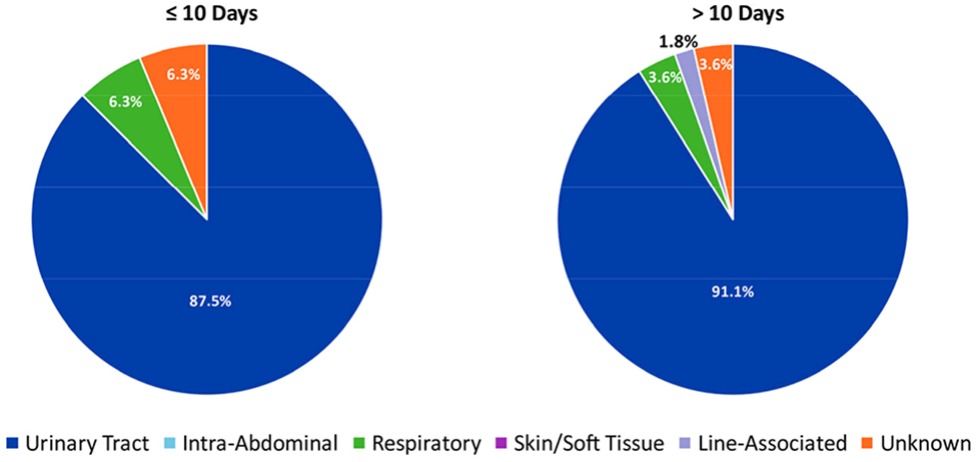
Source of infection

**Table 2:**
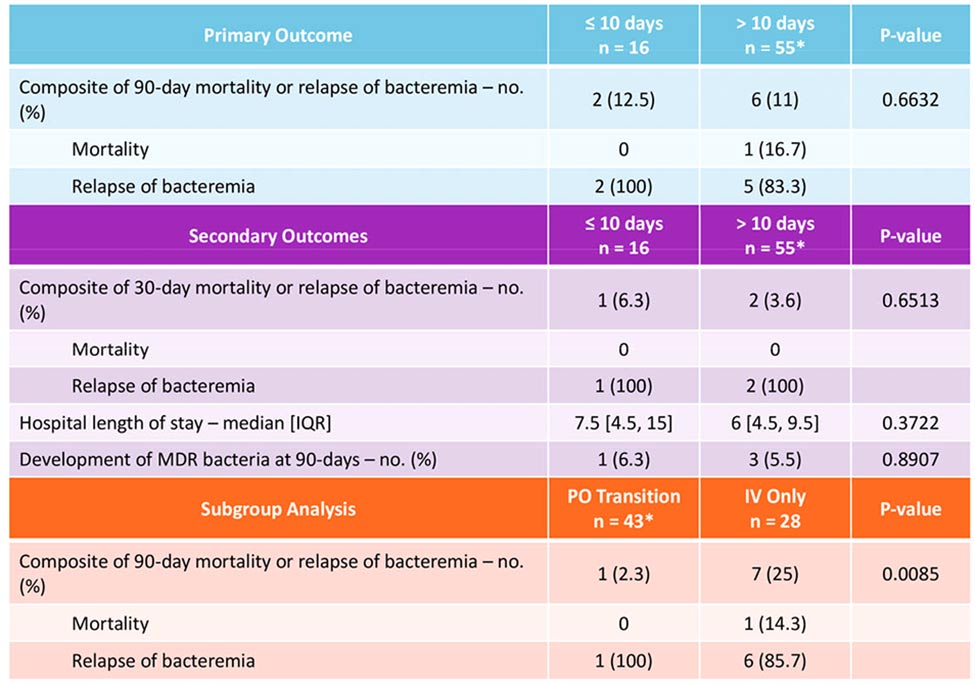
Primary and secondary outcomes *Excluded patients that could not be assessed

**Results:**

A total of 72 patients were included in the study. Sixteen patients received ≤ 10 days and 56 received > 10 days of antibiotics with a mean duration of 9 and 14 days respectively. The primary composite of 90-day mortality or relapse of bacteremia was not statistically significant at 12.5% in the ≤ 10-day group and 11% in the > 10-day group. There were no statistically significant differences in any secondary outcomes. In our subgroup analysis, patients remaining on IV antibiotics had higher rates of 90-day mortality or relapse at 14.3% vs. 2.3% in those transitioned to PO antibiotics (p = 0.0085).

**Conclusion:**

This study demonstrates that prescribing patterns for treatment of Gram-negative bacteremia in KTRs vary. Those who received ≤ 10 days had similar outcomes compared to those who received > 10 days. Shorter courses may be adequate for Gram-negative bacteremia in KTRs, however larger studies are required to confirm these results.

**Disclosures:**

All Authors: No reported disclosures

